# B Cell Receptor Signaling-Based Index as a Biomarker for the Loss of Peripheral Immune Tolerance in Autoreactive B Cells in Rheumatoid Arthritis

**DOI:** 10.1371/journal.pone.0102128

**Published:** 2014-07-24

**Authors:** Taras Lyubchenko, Gary O. Zerbe

**Affiliations:** 1 Division of Rheumatology, University of Colorado School of Medicine, Aurora, Colorado, United States of America; 2 Department of Biostatistics and Informatics, University of Colorado School of Public Health, Aurora, Colorado, United States of America; University Hospital Jena, Germany

## Abstract

This study examines the loss of peripherally induced B cell immune tolerance in Rheumatoid arthritis (RA) and establishes a novel signaling-based measure of activation in a subset of autoreactive B cells - the *Induced tolerance status index* (ITSI). Naturally occurring naïve autoreactive B cells can escape the “classical” tolerogenic mechanisms of clonal deletion and receptor editing, but remain peripherally tolerized through B cell receptor (BCR) signaling inhibition (postdevelopmental “receptor tuning” or anergy). ITSI is a statistical index that numerically determines the level of homology between activation patterns of BCR signaling intermediaries in B cells that are either tolerized or activated by auto antigen exposure, and thus quantifies the level of peripheral immune tolerance. The index is based on the logistic regression analysis of phosphorylation levels in a panel of BCR signaling proteins. Our results demonstrate a new approach to identifying autoreactive B cells based on their BCR signaling features.

## Introduction

We recently reported about changes in B cell antigen receptor signaling profiles associated with the loss of postdevelopmentally induced peripheral immune tolerance (anergy) in a subset of autoreactive B cells in patients with Rheumatoid arthritis [1], [2]. This follow-up study further examines the loss of B cell immune tolerance in RA and proposes a novel BCR signaling-based measure of autoimmune activation in the autoreactive B cell subset - the *Induced Tolerance Status Index* (ITSI).

Significant evidence supports the pathogenic role of autoreactive B cells in Rheumatoid arthritis (RA) [3], [4], [5], [6], [7], [8]. B cell targeted therapies are effective in RA (reviewed in [6], [9], [10]). B cells accumulating in the joints of RA patients produce autoreactive antibodies [11] and elevated B cell antigen receptor (BCR) signaling activity positively correlates with RA assessment scores [12]. The loss of B cell immune tolerance has been recognized as an important factor in the onset of RA [7], [8], [13].

BCR signaling regulation mechanisms involved in the control of peripheral immune tolerance have been under investigation from the autoimmunity perspective since the development of suitable transgenic mouse models in the 1990's [14], [15], [16]. The postdevelopmentally induced peripheral immune tolerance is attained through selective BCR signaling inhibition (receptor tuning, anergy [17], [18], [19]) in naturally occurring autoreactive B cells that have escaped the “classical” tolerogenic mechanisms of clonal deletion and receptor editing [20], [21] earlier in the development. These B cells recognize self-antigens and have the capacity to produce autoreactive antibodies, but under normal conditions are prevented from autoimmune activation by selective intrinsic BCR signaling inhibition [22]. However, this potentially autoreactive B cell subset remains vulnerable to activation by crossreactive autoantigens [23]. Peripheral induced immune tolerance is a signaling phenomenon, and the tolerogenic balance between activatory and inhibitory BCR signaling inputs is reversible [24], [25].

**Figure 1:**
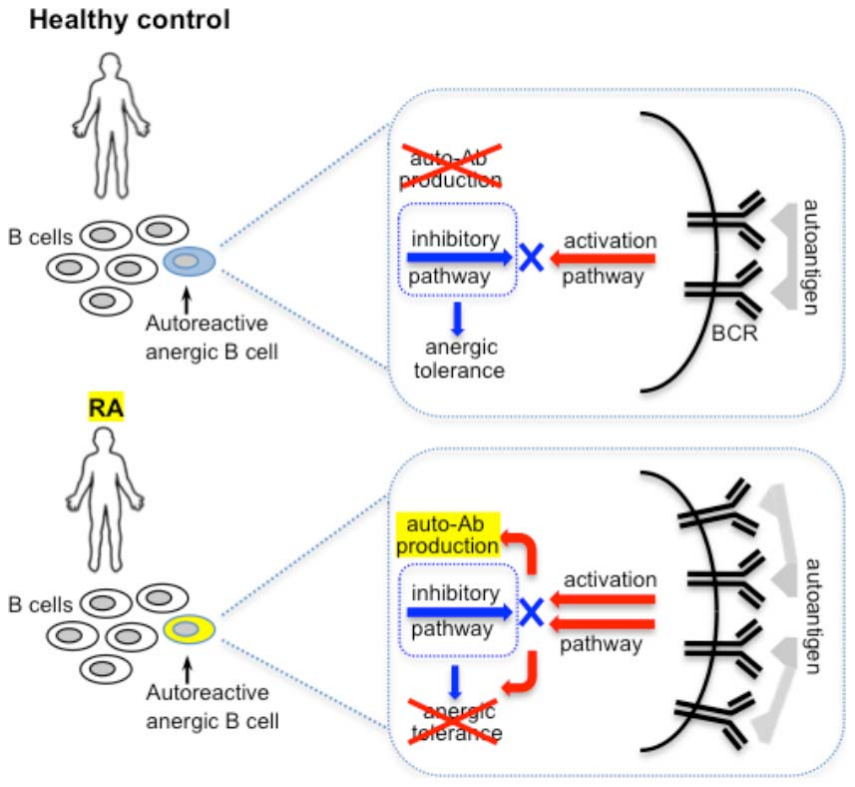
Normal (tolerance in healthy controls) and pathological (broken tolerance in RA) BCR signaling in autoreactive B cells with induced peripheral immune tolerance.

Despite the landmark studies in mouse models [23], [26], a distinct subset of naturally occurring autoreactive B cells with postdevelopmentally induced tolerance (**PIT** for short) has been identified in humans only recently [27]. These naïve CD19^+^CD27^−^IgD^+^IgM^low/−^ peripheral blood B cells (termed **B_ND_** [27]) retain a degree of autoreactivity after receptor editing, are hyporesponsive to BCR stimulation *in vitro* and produce autoreactive Abs (anti-dsDNA and anti-HEp-2). This autoreactivity appears innate, as variable *Ig* genes show no evidence of somatic hypermutation, indicating that it was not generated during an adaptive immune response. Two major signaling properties distinguish cells with PIT from other B cell types: reduced amplitude of protein phosphorylation and Ca^2+^ responses to antigen receptor ligation, and elevated BCR-related baseline protein phosphorylation activity in intact cells. These signaling features, described in both humans and mice [17], [23], [27], reflect chronic stimulation with crossreactive autoantigens paralleled by the continuous inhibition of intracellular BCR signaling through downregulatory mechanisms, and represent an important mechanism that prevents autoimmunity (Figure 1).

After the recent definitive reports on the postdevelopmantally tolerized autoreactive B_ND_ cell subset in humans [2], [27], [28], new evidence has emerged suggesting that PIT sustained via tolerogenic BCR signaling inhibition is present in a much larger fraction of human B cells than previously thought, and this reflects a state of peripheral immune tolerance induced by the chronic autoantigen stimulation [28]. Recent studies in RA patients also revealed the increased frequency of B cells with PIT producing germline autoreactive antibodies, which recognize nuclear and cytoplasmic structures [29]. Elevated numbers of highly autoreactive B cells with PIT found in the blood of RA patients are not being eliminated, and remain in circulation for extended periods, which creates a favorable environment for breaking the tolerance in this B cell subset [29]. Furthermore, it has been demonstrated that in contrast to T cells, immune tolerance attained through BCR signaling inhibition, rather than clonal deletion, accounts for a very significant part of B cell tolerance, and that antigen exposure “tunes” the responsiveness of BCR signaling in B cells by downmodulating the expression of surface IgM and by modifying basal Ca^2+^ levels, and that a continuum of functional PIT persists in the mature naïve B cell repertoire [30]. According to recent assessments, it is clear that postdevelopmental B cell tolerogenic mechanisms of selective BCR signaling inhibition are compromised in the onset of autoimmunity (reviewed in [31]).

**Figure 2:**
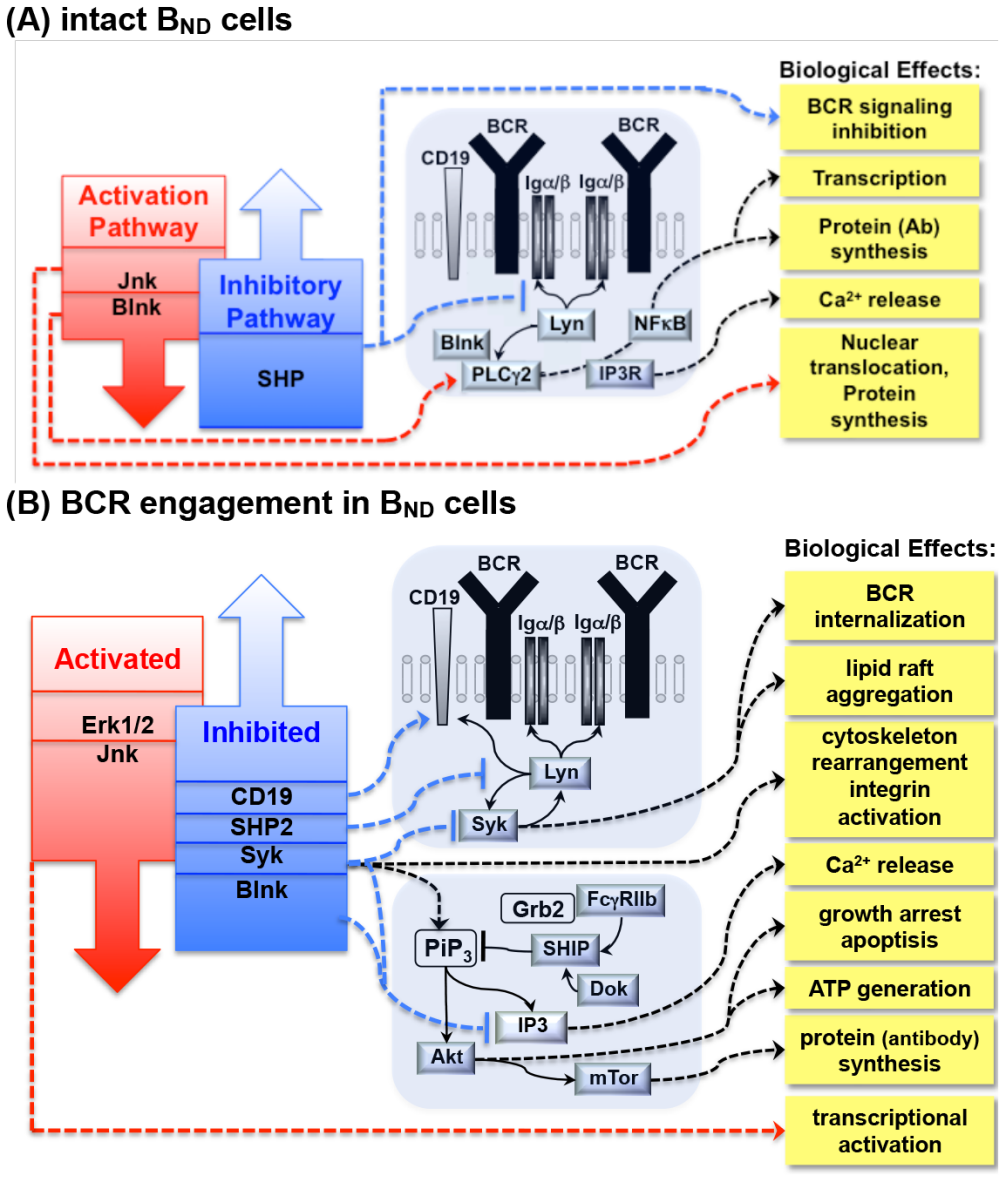
BCR signaling pathways associated with the loss of peripheral induced tolerance in autoreactive B_ND_ cells of RA patients [2]. (A) Unmanipulated B_ND_ cells: increased baseline activity Blnk, SHP and Jnk. **(B)** Response to BCR engagement in B_ND_ cells: decreased phosphorylation of Blnk, Syk, SHP2, CD19 and increased activation of Erk1/2, Jnk.

Studies in our laboratory have established the loss of PIT in autoreactive B_ND_ cells as a contributing factor in the development of RA and demonstrated that inhibitory BCR signaling cascades involved in the maintenance of PIT are altered in human RA [2], as well as in the mouse model of experimentally induced arthritis [32]. Major biological effects associated with the loss of tolerogenic BCR signaling inhibition in human B_ND_ cells in RA are illustrated in Figure 2 in the context of signaling pathways. In addition, our studies in human subjects discovered significant positive correlations between BCR signaling activity in B_ND_ cells and RA Clinical Disease Activity Index (CDAI) [33], and established phosphorylation levels of specific BCR signaling intermediaries as predictors of RA through linear regression analysis [2]. These findings demonstrated a link between phosphoprotein signaling activity in autoreactive anergic B cells and clinical manifestations of RA. Noteworthy, RA-related phosphoprotein profile changes are unique to the CD19^+^CD27^−^IgD^+^IgM^low/−^ subset of autoreactive B_ND_ cells: nominal logistic fit demonstrated that RA predictive value of the combined phosphorylation profile of several major BCR signaling proteins and Ca^2+^ levels in response to BCR stimulation [2] in B_ND_ cells was highly significant (R^2^ = 1.0, p = 0.0002), while in mature naïve CD19^+^IgM^+^ B cells this predictive value was substantially less evident (R^2^ = 0.505, p = 0.053). This moderate effect in CD19^+^IgM^+^ cells may be related to the recently recognized immunoregulatory role of B cells with PIT [34].

Linear regression analysis of the combined group of RA patients and normal controls, adjusted for the subject's age and gender, demonstrated a modest trend toward a lower total tyrosine phosphorylation activity in response to BCR cross-linking with age (R^2^ = 0.12, p = 0.0518). However, RA subjects exhibited substantially increased phosphoprotein responses to BCR stimulation in the CD19^+^CD27^−^IgD^+^IgMl^low/−^ B cell subset, as compared to normal controls (total pTyr mean±SE % increase of 121.4±30.6 vs. 22.5±31.0 in RA and control groups, respectively (p = 0.05)), suggesting that the breach of PIT in RA patients occurs regardless of the age (data previously reported [2]).

## Results and Methods

**Figure 3:**
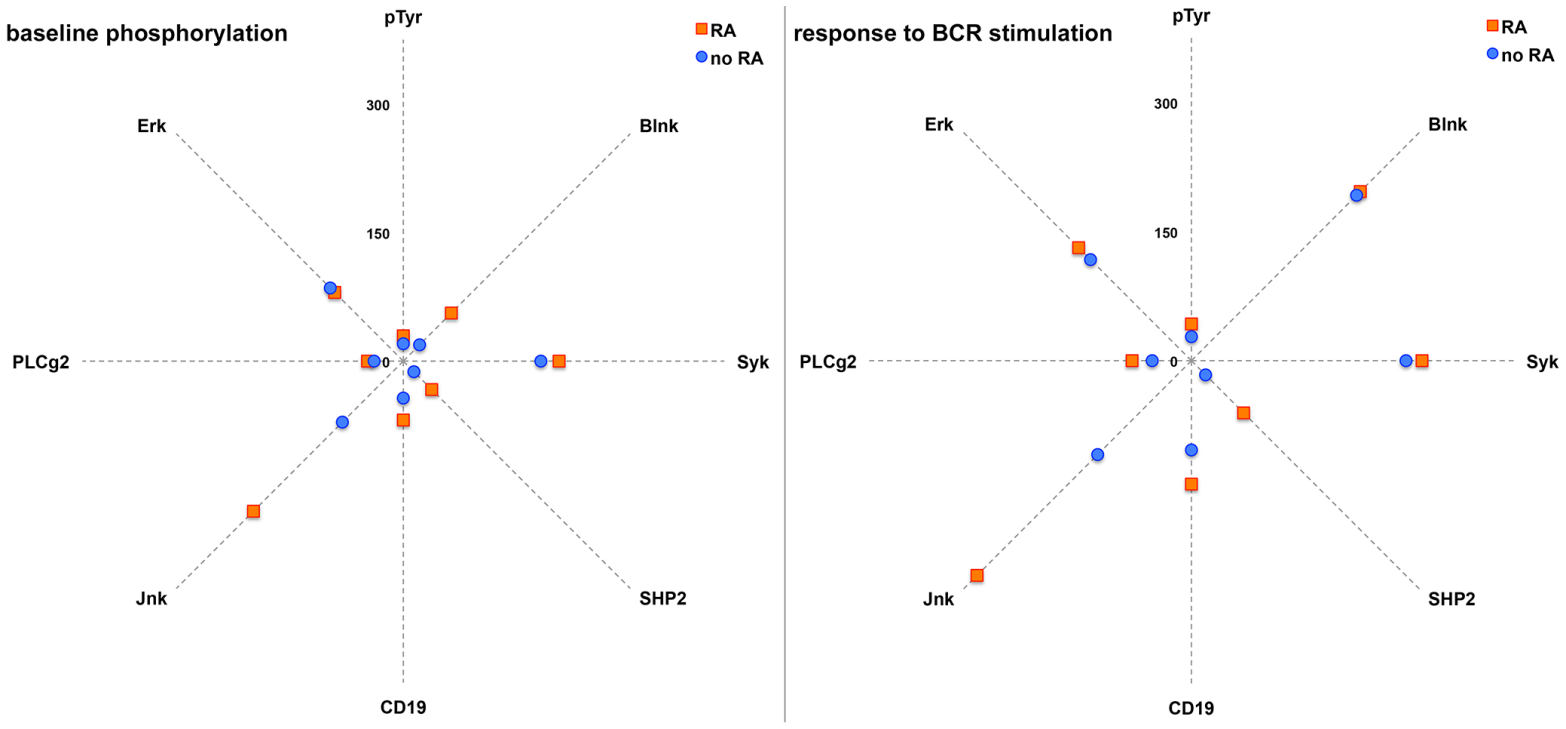
Phosphorylation levels of major BCR signal transduction proteins and total tyrosine phosphorylation levels (pTyr) in CD27^−^IgD^+^IgM^low/−^ B cells of RA patients (red) and healthy control subjects (blue) at baseline and in response to anti-BCR stimulation *in vitro*.

Inhibitory signaling features that maintain PIT in B_ND_ cells of healthy controls are reversed/altered in RA and B_ND_ cells isolated from RA patients exhibit distinctly different phosphorylation (activation) patterns of major BCR signaling intermediaries [2]. These findings are based on the analysis of BCR signaling protein phosphorylation profiles of 32 healthy controls (mean age 35.7; male/female ratio 0.68) and 20 RA patients (mean age 55.9; male/female ratio 0.54) recruited at the University of Colorado Rheumatology Clinic. RA subjects have been diagnosed in recent years, were RF/anti-CCP positive, treated with DMARDs and have not undergone steroid or B cell targeted treatments. Subjects were recruited according to the Colorado Institutional Review Board approved protocol (#10-0250) with a written informed consent agreement. PBMC were isolated from freshly collected blood samples (IV 20 mL). Phosphorylation levels of BCR signal transduction proteins were measured with BD Phosflow assay in CD19^+^CD27^−^IgD^+^IgM^low/−^ B_ND_ cells as described in [2]; cells were stimulated (or not) with anti-BCR (polyclonal goat F(ab′)2 anti-human Ig(H+L) purchased from Southern Biotech) *in vitro* for 10 min and baseline and phospho-response amplitude levels of individual BCR signal transduction proteins were compared between RA patients and healthy controls [2]. Figure 3 compares phosphorylation levels of major BCR signal transduction proteins and total tyrosine phosphorylation levels (pTyr) in CD19^+^CD27^−^IgD^+^IgM^low/−^ B_ND_ cells of RA patients (red) and healthy control subjects (blue) at baseline and in response to anti-BCR stimulation *in vitro* (as described in [2]). The integrated pattern of relative distances between blue and red dots for each BCR signaling protein illustrates differences in phosphoprotein activation levels for each group (i.e. red/blue dots would overlap if phosphoprotein activation levels were identical). These patterns appear different in unmanipulated B_ND_ cells (Figure 3, left panel), as well as in cells stimulated with anti-BCR (Figure 3, right panel).

### Induced Tolerance Status Index (ITSI)

To quantify the BCR signaling patterns (Figure 3) in order to establish a signaling-based measure of autoimmune activation in autoreactive B_ND_ cells, we propose the *Induced tolerance status index* (ITSI). This statistical parameter numerically determines the level of homology between BCR phosphoprotein activation patterns in B_ND_ cells (CD19^+^CD27^−^IgD^+^IgM^low/−^) with intact PIT (healthy controls) vs. the cells with compromised PIT (RA patients), and thus quantifies the level of PIT. The index is based on the logistic regression analysis of phosphorylation levels in the panel of B cell signaling proteins in individual subjects. Phosphorylation of specific BCR signaling proteins was assessed with BD Phosflow assay as mean fluorescence intensity (MFI) of protein-specific phospho-Ab staining as described [2]. BCR protein phosphorylation patterns (those exemplified in Figure 3) were analyzed by logistic regression in cross-referenced datasets of B_ND_ cells that were unmanipulated or stimulated with anti-BCR, and obtained from either RA patients or healthy control subjects [2].

### Statistical Analysis

This type of statistical approach (i.e. logistic regression) has been successfully used in another study in our laboratory to compute the Cytokine Score [35] in order to address a mathematically similar task of analyzing complex associations between cytokine production patterns and the risk of RA [35].

**Figure 4:**
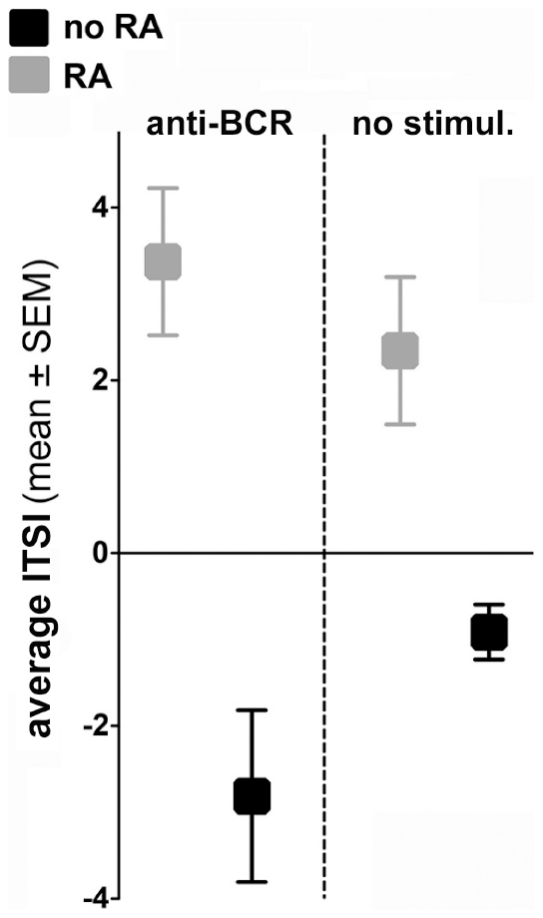
Induced tolerance status index (ITSI) discriminates between autoreactive B_ND_ cells from healthy controls and RA subjects based on BCR phosphoprotein activation patterns.

The current study compares ITSI parameters between human subjects with RA and healthy controls through the logistic regression analysis of our recently reported data [2] on 7 signaling proteins (Blnk, Syk, SHP2, CD19, Jnk, PLCγ2, Erk1/2) and total pTyr in B_ND_ cells with assumed dichotomous type of dependant variable (i.e. RA = Yes/No). Because the logistic regression analysis requires complete datasets, some subjects with missing data points for at least one of the phosphoproteins had to be excluded altogether (15 subjects with RA and 12 controls had complete datasets). We designed an algorithm (Appendix 1) to calculate logistic regression coefficients and to construct a composite index that could discriminate RA from healthy control subjects with sensitivity of 88.7% and specificity of 83.3% based on the phosphorylation pattern changes in response to anti-BCR stimulation, and with sensitivity of 73.3% and specificity of 83.3% based on the baseline protein phosphorylation levels. In both instances, the ITSI index predictably distinguished between RA and healthy control groups based on their B_ND_ BCR signaling profiles (Figure 4). ITSI ranged between −2.16 and 10.92 (RA) and −9.95 and 1.06 (no RA) for anti-BCR-stimulated B_ND_ cells, and from −1.82 to 11.42 (RA) and from −3.19 to 0.20 (no RA) for baseline MFI phosphorylation values. The mean value of the index ± standard error was 3.37±0.85 (RA) and −2.81±0.99 (no RA) for anti-BCR-stimulated B_ND_ cells, and 2.34±0.85 (RA) and −0.91±0.32 (no RA) for baseline MFI phosphorylation values (Figure 4). Data analysis algorithm (SAS code) is shown in Appendix 1.

Supplemental data in Data S1 and Data S2 show individual subject datapoins with spreads and further statistical analysis, and a detailed description of the logistic regression algorithm, including the model fit statistics, analysis of maximum likelihood estimates for each individual phosphoprotein in the pattern, sensitivity and specificity calculation tables, odds ratio estimates and associations of predicted probabilities with observed responses.

## Discussion

In RA, chronic exposure to the omnipresent autoantigens can set off B cell activation and production of pathogenic autoreactive Abs [36]. Our study examines statistical distributions of BCR phosphoprotein signaling patterns in autoreactive B_ND_ cells in patients with RA. Our recent studies identified unique BCR signaling features associated with the loss of immune tolerance in a subset of CD19^+^CD27^−^IgD^+^IgM^low/−^ anergic B cells (B_ND_) in RA patients [2]. Autoreactive properties of this naturally occurring B cell subset were recently discovered [27]. B_ND_ cells exhibit a specific type of reversible immune tolerance – the post-developmentally induced peripheral tolerance (PIT), which is attained through selective BCR signaling inhibition (receptor tuning, anergy [17], [18], [19]) in a subset of autoreactive cells that escaped the central tolerogenic mechanisms of clonal deletion and receptor editing [20], [21] earlier in their development.

We have established the loss of PIT in autoreactive B_ND_ cells as a contributing factor in the development of RA and demonstrated that inhibitory BCR signaling cascades involved in the maintenance of PIT are altered in RA [2]. According to the central hypothesis of our study (Figure 1), in healthy individuals, autoreactive B_ND_ cells that have escaped the mechanisms of central tolerance maintain the peripheral immune tolerance (PIT) to ubiquitous autoantigens through continuous receptor tuning and BCR signaling inhibition, and our results demonstrate that this tolerogenic mechanism is compromised in RA.

PIT is a signaling phenomenon occurring late in the development cycle of B cells. However, the signaling aspects of this tolerogenic BCR signaling inhibition mechanism have not been studied in detail beyond the general features such as BCR-mediated Ca^2+^ influx and total protein phosphorylation activity (pTyr), particularly in human B cells. The novelty of our study is in addressing the signaling pathways involved in the maintenance and pathological alterations of PIT in RA with a quantitative approach. ITSI values provide a scale to measure the level of BCR signaling inhibition which sustains the postdevelopmentally induced peripheral immune tolerance and enables future studies of correlations between ITSI and clinical RA scores in large datasets generated by comprehensive phosphoprotein microarrays. Our preliminary findings in a relatively small 8-phosphoprotein panel are encouraging. Furthermore, although murine models of autoimmunity provided important insights into peripheral tolerogenic mechanisms in B cells and suggested that the loss of PIT has clinical significance in autoimmunity, human autoreactive B cells with PIT have not been well studied due to the difficulty of identifying this subset. Recent discovery of the B_ND_ subset in humans [27] provided an opportunity to study the role of PIT in RA (and other inflammatory autoimmune diseases where B cells play a pathogenic role). To our knowledge, our study is [among] the first to address the tolerogenic/inhibitory BCR signaling mechanisms in human B cells and investigate the loss of peripherally induced immune tolerance in RA with statistical analysis of BCR signaling patterns.

In our previous studies of RA, the conclusions about specific BCR signaling pathways involved in the alterations of PIT were based on direct comparisons of baseline phosphoprotein activation levels and normalized anti-BCR response amplitudes in the context of signaling cascades in RA vs. control groups and on the empirical analysis of phosphorylation/activation levels of specific BCR signaling proteins based on their known functions in the BCR pathways (i.e. Figure 2), as well as on pairwise correlation matrices of the activation of levels of phosphoproteins involved in BCR signal transduction, and on correlations between the BCR signaling activity in B_ND_ cells and severity of RA clinical manifestations [2]. These approaches are summarized in Table 1A–C. ITSI, however, enables the integrative quantification of these differences between BCR phosphoprotein activation patterns through logistic regression analysis (Table 1D). ITSI predictably discriminates between autoreactive B_ND_ cells that either have (control group) or do not have (RA group) the PIT. Authors believe this report will be of interest as it demonstrates a new approach to identify autoreactive B cells based on their BCR signaling features, and introduces a novel signaling-based biomarker associated with the loss of B cell immune tolerance in inflammatory autoimmune diseases such as Rheumatoid arthritis.

**Table 1:** Strategies for BCR phosphoprotein data analysis in autoreactive B_ND_ cells.

Data analysis approach			
Phosphoprotein activation patterns in B_ND_ cells with postdevelopmentally induced tolerance (PIT)			**D.** BCR signaling-based ***Induced Tolerance Status Index*** (ITSI) as a functional biomarker of autoimmune activation.
**A.** Direct comparison of normalized anti-BCR response amplitudes in the context of signaling cascades in RA vs. control groups.	**B.** Pairwise correlation matrix of the activation levels of phosphoproteins involved in BCR signal transduction.	**C.** Correlation between BCR signaling activity in B_ND_ cells and severity of RA.	
*in vitro* B cell responses to anti-BCR calculated as % increase over corresponding non-stimulated controls separately for B_ND_ and CD19^+^IgM^+^ B cell subsets for each subject, followed by the calculation of relative differences between % response amplitudes of B_ND_ and CD19^+^IgM^+^ B cells within RA and control groups independently.	Spearman's multivariate analysis to establish correlation-based pairwise relationships among BCR signaling intermediaries in B_ND_ cells. Correlation matrices for phosphoprotein activation levels in control and RA groups.	1) Logistic fit model to establish the overall signaling activity in B_ND_ cells as a predictor of RA. 2) Linear regression model to correlate the activity of individual BCR signal transduction proteins with RA severity score (CDAI, DAS28crp).	Logistic regression analysis computes ITSI - a statistical parameter that numerically determines the level of homology between BCR phosphoprotein activation patterns in B_ND_ vs. naïve CD19^+^IgM^+^ B cells, and thus quantifies the level of PIT.

Results and interpretation			
1) Determine changes in phospho-rylation of each BCR signaling protein associated with RA. 2) Direct comparison between phosphoprotein signaling patterns in B_ND_ cells in control and RA subjects. 3) Empirical conclusions on the balance of stimulatory and inhibitory BCR signaling pathways in B_ND_ cells based on the known functions of these proteins in BCR pathways. 4) Predictions on the physiological outcomes of B cell activation based on the known BCR pathway roles.	1) Statistically significant correlation values indicate that increases/decreases in the phosphorylation of each protein pair are pairwise linked and therefore the two proteins are functionally involved in the same signaling pathway. 2) Correlations between proteins involved in multiple or different signaling pathways reveal the interpathway crosstalk. 3) Establish a detailed PIT-specific phosphorylation pattern for proteins involved in BCR signaling inhibition in B_ND_ cells, and thus define the relationship between stimulatory and inhibitory signaling pathways.	1) Association between the global BCR signaling activity in B_ND_ cells and severity of RA. 2) Determine that RA-related BCR phospho-protein profile changes are unique to the B_ND_ cell subset. 3) Signaling-based biomarker approach to identify phosphoprotein predictors of RA based on the activation of autoreactive B_ND_ cells.	1) Lower ITSI values reflect increased levels of immune tolerance (PIT) and suppressed responses to autoantigens in B_ND_ cells. 2) ITSI discriminates between control and RA groups based on B_ND_ BCR signaling patterns. 3) Propose the ITSI as a signaling-based diagnostic biomarker for the loss of peripheral tolerance in autoreactive B_ND_ cells.

The association of the HLA region with RA was noted when the frequency of individuals with the HLA-Dw4 serotype was found to be increased among RA patients compared with healthy controls [37]. This particular serotype links a set of alleles at the HLA-DR gene. Many studies have examined and expanded these associations in order to better elucidate the genetic underpinnings of rheumatoid arthritis. Both linkage and association studies of the HLA-DR gene have confirmed that it is a genetic susceptibility locus for RA and provided an important clue to pathogenesis. Subsequent discoveries using high-throughput genotyping have identified over 40 additional loci outside of the HLA locus that also play roles in RA risk and implicate various pathways in pathogenesis. However, the issue of clinical utility of genotyping in patients with RA remains unresolved (reviewed in [38], [39], [40], [41], [42]). HLA genotyping/matching of control and RA groups were not performed in our study because we have no direct evidence that known HLA polymorphisms that may (or may not) be involved in antigen-specific signaling in T cells, can have an effect on intrinsic BCR signaling events that are not the result of adaptive (antigen specific) B cell immune response [27], which is the major focus of our study. While the subjects in our study were not HLA-matched, the ITSI score clearly discriminated between RA vs. non-RA groups based on BCR phosphoprotein activation patterns that reflect the loss of peripherally induced immune tolerance. However, the importance of HLA-related genetic susceptibility factors in RA should not be underestimated, as little is known about the role of these factors in the peripherally induced B cell tolerance. Although beyond the focus of this report, potential associations between specific HLA phenotypes and B cell immune tolerance in RA patients are a promising subject for future studies.

Our initial findings strongly support the prospective use of ITSI as a clinically relevant diagnostic measure of the humoral immunity/B cell component in RA, particularly in light of the recent evidence suggesting that peripherally induced immune tolerance in response to the chronic autoantigen stimulation is present in a major fraction of human naive B cells, and the scale of this tolerogenic mechanism is unique to B cells, and that peripheral tolerogenic mechanisms of selective BCR signaling inhibition are compromised in the onset of autoimmunity [28], [30], [31]. Building up on our initial findings in the relatively small 8-phosphoprotein panel used in our pilot study [2], clinical relevance of the ITSI score will benefit from further validation in additional RA patient subgroups with the use of a comprehensive B cell phosphoprotein array. Results of our studies in RA patients indicate that BCR signal inhibitory pathways that maintain peripherally induced B cell tolerance are altered in RA and provide the rationale for future studies to investigate the loss of B cell anergic tolerance as a contributing factor in the pathigenesis of the disease. This approach will likely identify BCR signaling proteins that can be targeted for pharmacological interference, and used as signaling-based functional biomarkers associated with the loss of peripheral B cell tolerance in RA. The results will provide new insights into the role of B cell tolerance in the pathology and early diagnosis of RA.

In addition to the CD19^+^CD27^−^IgD^+^IgM^low/−^ subset of autoreactive B_ND_ cells, recent reports suggest that another B cell subset with anergic features – IgM^+^CD21^low^ may also play a role in autoimmunity. According to recent reports [29], [43], this subset comprises a minor fraction (under 1%) of the “bulk” of mature naïve CD19^+^CD27^−^IgM^+^CD21^+^ peripheral blood B cells, but may expand by a few percentage points in some (but not all) RA patients [29]. However, the pathogenic role the IgM^+^CD21^low^ B cell subset has not been definitively characterized after the initial reports that indicated its potential involvement in RA and in hepatitis C virus infection-related autoimmunity. IgM^+^CD21^low^ anergic B cells are phenotypically different from the IgM^−/low^ B_ND_ subset. Recent evaluations suggest that RA is best considered as a clinical syndrome spanning several disease subsets [36]. These different subsets involve a number of inflammatory cascades that all lead towards a final common pathway with persistent synovial inflammation and associated damage to the cartilage, and engage multiple B cell subsets that may include more than one type of anergic B cells. IgM^+^CD21^low^ anergic B cells are a promising target for the investigation of their pathogenic role in RA, however, such studies constitute a separate research direction and are beyond the main focus of this study - the IgM^−/low^ B_ND_ cells.

## Conclusions

1. Our results demonstrate a novel approach for identifying autoreactive B cells based on intrinsic BCR signaling features and introduce the ITSI score as signaling-based biomarker associated the loss of peripheral immune tolerance in the autoreactive B cell subset in RA.
2. ITSI discriminates between control and RA groups based on the inhibitory BCR phosphoprotein signaling patterns in autoreactive B cells, and thus confirms the loss of induced peripheral immune tolerance as a pathogenic factor in RA.
3. Lower ITSI values reflect increased levels of peripheral immune tolerance and suppressed responses to autoantigens in B_ND_ cells.
4. We recommend ITSI as a potential diagnostic measure of activation for innately autoreactive B_ND_ cells in RA.

## Supporting Information

Appendix S1 **Logistic regression algorythm (SAS code).**
(PDF)

Data S1 **Individual subject datapoins with spreads and basic statistical analysis.**
(PDF)

Data S2 **Detailed description of the logistic regression analysis with model fit statistics, maximum likelihood estimates for each individual phosphoprotein in the pattern, sensitivity and specificity calculation tables, odds ratio estimates and associations of predicted probabilities with observed responses.**
(PDF)
