# Supplementary material for: B Cell Receptor Signaling-Based Index as a Biomarker for the Loss of Peripheral Immune Tolerance in Autoreactive B Cells in Rheumatoid Arthritis
Source: PLoS One. 2014 Jul 24;9(7):e102128. doi: 10.1371/journal.pone.0102128 (PMC4109936; doi:10.1371/journal.pone.0102128)
Supplement: Appendix S1 — Logistic regression algorythm (SAS code). (PDF) [file pone.0102128.s001.pdf]

Appendix S1.
SAS Code

```
options nocenter;
PROC IMPORT OUT= WORK.RA-VS-CONTROL
            DATAFILE= "C:\Ddrive\People\GG-
MM\LL\Lyubchenko\16Sept\G
Z ALL.xlsx"
            DBMS=EXCEL REPLACE;
     RANGE="Sheet1$";
     GETNAMES=YES;
     MIXED=NO;
     SCANTEXT=YES;
     USEDATE=YES;
     SCANTIME=YES;
     /*  Obs ID RA pTyrB pTyrS pBlnkB pBlnkS pSykB pSykS pSHP2B pSHP2S
pCD19B pCD19S pJnkB pJnkS pPLCg2B pPLCg2S pErkB pErkS   */
RUN;

data RA-VS-CONTROL;  set RA-VS-CONTROL;
  IndexS= -2.1246+0.00794*pBlnkS-
0.00945*pSykS+0.1427*pSHP2S+0.0179*pCD19S+0.00530*pJnkS-
0.1646*pPLCg2S+0.00835*pErkS;
  if IndexS>0 then PRAs='y';  if IndexS<=0 then PRAs='n';  if IndexS=.
then PRAs ='  ';
  IndexB=-1.0912+0.0160*pBlnkB+0.00130*pSykB+0.0754*pSHP2B-
0.0309*pCD19B+0.0124*pJnkB-0.0327*pPLCg2B-0.00491*pErkB;
  if IndexB>0 then PRAb='y';  if IndexB<=0 then PRAb='n';  if IndexB=.
then PRAb ='  ';
run;

title 'MFI after stimulation';
proc print data=RA-VS-CONTROL nobs;   var ID RA  pTyrS  pBlnkS  pSykS
pSHP2S  pCD19S  pJnkS  pPLCg2S  pErkS IndexS PRAs;  run;
proc logistic;  model RA(event='y') = pBlnkS  pSykS  pSHP2S  pCD19S
pJnkS  pPLCg2S  pErkS;   run;
proc freq; table RA*PRAs;  run;

title 'MFI baseline';
proc print data=RA-VS-CONTROL nobs;   var ID RA  pTyrb  pBlnkb  pSykb
pSHP2b  pCD19b  pJnkB  pPLCg2B  pErkB Indexb PRAb;  run;
proc logistic;  model RA(event='y') = pBlnkB  pSykB  pSHP2B  pCD19B
pJnkB  pPLCg2B  pErkB;   run;
proc freq; table RA*PRAb;  run;
```
