# Supplementary material for: B Cell Receptor Signaling-Based Index as a Biomarker for the Loss of Peripheral Immune Tolerance in Autoreactive B Cells in Rheumatoid Arthritis
Source: PLoS One. 2014 Jul 24;9(7):e102128. doi: 10.1371/journal.pone.0102128 (PMC4109936; doi:10.1371/journal.pone.0102128)
Supplement: Data S1 — Individual subject datapoins with spreads and basic statistical analysis. (PDF) [file pone.0102128.s002.pdf]

Supplement 1

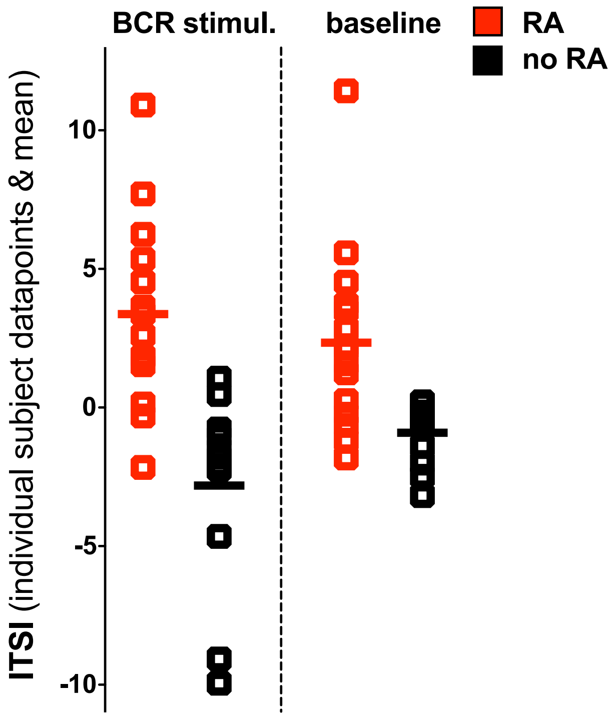

	RA BCR stimul.	noRA BCR stimul	RA baseline	noRA baseline
**Mean** ± SEM	**3.3741** ± 0.8527	**-2.8125** ± 0.9945	**2.3435** ± 0.8529	**-0.91354** ± 0.3180
**t test (P value)**	0.0014	0.0164	0.0157	0.0152
Minimum	-2.1573	-9.9477	-1.8167	-3.186
25% Percentile	1.546	-4.0465	-0.2418	-1.8544
Median	3.3853	-1.9449	2.1431	-0.48605
75% Percentile	5.348	-0.8438	3.7232	-0.17788
Maximum	10.916	1.0577	11.423	0.2002
