# Supplementary material for: B Cell Receptor Signaling-Based Index as a Biomarker for the Loss of Peripheral Immune Tolerance in Autoreactive B Cells in Rheumatoid Arthritis
Source: PLoS One. 2014 Jul 24;9(7):e102128. doi: 10.1371/journal.pone.0102128 (PMC4109936; doi:10.1371/journal.pone.0102128)
Supplement: Data S2 — Detailed description of the logistic regression analysis with model fit statistics, maximum likelihood estimates for each individual phosphoprotein in the pattern, sensitivity and specificity calculation tables, odds ratio estimates and associations of predicted probabilities with observed responses. (PDF) [file pone.0102128.s003.pdf]

Supplement 2

IndexS = Protein Index after stimulation;

PRAs = predicted RA based on this index.

Table 1 reports the results of a logistic regression of RA on all protein phosphorylation values (MFI) after stimulation.

The protein index after stimulation is a linear combination of the proteins weighted by the regression coefficients in Table 1.

Predicted RA is based on this index.

Table 2 is a classification table of RA versus Predicted RA. Sensitivity and specificity of predicted RA for RA are reported below.

Tables 3, 4 are a repeat of Tables 1, 2, but for baseline protein MFI values.

The Appendix1 lists the SAS code used to create these tables.

MFI after stimulation

The LOGISTIC Procedure

Model Information		
**Data Set**	WORK.RA-VS-CONTROL	
**Response Variable**	RA	RA
**Number of Response Levels**	2	
**Model**	Binary logit	
**Optimization Technique**	Fisher's scoring	

Probability modeled is RA='y'.

Model Convergence Status
Convergence criterion (GCONV=1E-8) satisfied.

Model Fit Statistics

Criterion	Intercept Only	Intercept and Covariates
**AIC**	39.096	32.228

Model Fit Statistics		
**Criterion**	**Intercept Only**	**Intercept and Covariates**
**SC**	40.392	42.595
**-2 Log L**	37.096	16.228

Testing Global Null Hypothesis: BETA=0			
**Test**	**Chi-Square**	**DF**	**Pr > ChiSq**
**Likelihood Ratio**	20.8678	7	0.0040
**Score**	13.6906	7	0.0570
**Wald**	6.3873	7	0.4953

Table 1. Logistic Regression Results.
Analysis of Maximum Likelihood Estimates

Parameter	DF	Estimate	Standard Error	Wald Chi-Square	Pr > ChiSq
**Intercept**	1	-2.1246	1.4759	2.0724	0.1500
**pBlnkS**	1	0.00794	0.00614	1.6688	0.1964
**pSykS**	1	-0.00945	0.00623	2.2994	0.1294
**pSHP2S**	1	0.1427	0.0640	4.9688	0.0258
**pCD19S**	1	0.0179	0.0143	1.5745	0.2096
**pJnkS**	1	0.00530	0.00372	2.0281	0.1544
**pPLCg2S**	1	-0.1646	0.0781	4.4390	0.0351
**pErkS**	1	0.00835	0.00888	0.8853	0.3468

```
IndexS= -2.1246+0.00794*pBlnkS-0.00945*pSykS+0.1427*pSHP2S+0.0179*pCD19S
                +0.00530*pJnkS-0.1646*pPLCg2S+0.00835*pErkS;

if IndexS>0 then PRAs='y';  if IndexS<=0 then PRAs='n';  if IndexS=. then PRAs ='  ';
```

Odds Ratio Estimates			
**Effect**	**Point Estimate**	**95% Wald Confidence Limits**	
**pBlnkS**	1.008	0.996	1.020
**pSykS**	0.991	0.979	1.003
**pSHP2S**	1.153	1.017	1.308
**pCD19S**	1.018	0.990	1.047
**pJnkS**	1.005	0.998	1.013
**pPLCg2S**	0.848	0.728	0.989
**pErkS**	1.008	0.991	1.026

Association of Predicted Probabilities and Observed Responses			
**Percent Concordant**	93.3	**Somers' D**	0.872
**Percent Discordant**	6.1	**Gamma**	0.877
**Percent Tied**	0.6	**Tau-a**	0.447
**Pairs**	180	**c**	0.936

MFI after stimulation

The FREQ Procedure

**Frequency**
**Percent**
**Row Pct**
**Col Pct**

Table 2 of RA by PRAs			
**RA(RA)**	**PRAs**		
	**n**	**y**	**Total**
**n**	37.04	7.41	44.44
	83.33	16.67	
	83.33	13.33	
**y**	7.41	48.15	55.56

	13.33	86.67	
	16.67	86.67	
**Total**	44.44	55.56	100.00

Sensitivity = 86.67%

Specificity = 83.33%

MFI baseline

The LOGISTIC Procedure

**Model Information**		
**Data Set**	WORK.RA-VS-CONTROL	
**Response Variable**	RA	RA
**Number of Response Levels**	2	
**Model**	binary logit	
**Optimization Technique**	Fisher's scoring	

Probability modeled is RA='y'.

**Model Convergence Status**
Convergence criterion (GCONV=1E-8) satisfied.

Model Fit Statistics

**Criterion**	**Intercept Only**	**Intercept and Covariates**
**AIC**	39.096	39.715
**SC**	40.392	50.082

Model Fit Statistics		
**Criterion**	**Intercept Only**	**Intercept and Covariates**
**-2 Log L**	37.096	23.715

Testing Global Null Hypothesis: BETA=0			
**Test**	**Chi-Square**	**DF**	**Pr > ChiSq**
**Likelihood Ratio**	13.3808	7	0.0634
**Score**	9.2290	7	0.2366
**Wald**	5.4277	7	0.6079

Table 3. Logistic Regression Results

Analysis of Maximum Likelihood Estimates

Parameter	DF	Estimate	Standard Error	Wald Chi-Square	Pr > ChiSq
**Intercept**	1	-1.0912	0.7978	1.8708	0.1714
**pBlnkB**	1	0.0160	0.0187	0.7312	0.3925
**pSykB**	1	0.00130	0.00433	0.0899	0.7643
**pSHP2B**	1	0.0754	0.0538	1.9667	0.1608
**pCD19B**	1	-0.0309	0.0308	1.0067	0.3157
**pJnkB**	1	0.0124	0.00845	2.1638	0.1413
**pPLCg2B**	1	-0.0327	0.0228	2.0609	0.1511
**pErkB**	1	-0.00491	0.00624	0.6198	0.4311

```
IndexB=-1.0912+0.0160*pBlnkB+0.00130*pSykB+0.0754*pSHP2B-0.0309*pCD19B
                  +0.0124*pJnkB-0.0327*pPLCg2B-0.00491*pErkB;

if IndexB>0 then PRAb='y';  if IndexB<=0 then PRAb='n';  if IndexB=. then PRAb ='  ';
```

Odds Ratio Estimates		
**Effect**	**Point Estimate**	**95% Wald Confidence Limits**

Odds Ratio Estimates			
**Effect**	**Point Estimate**	**95% Wald Confidence Limits**	
**pBlnkB**	1.016	0.980	1.054
**pSykB**	1.001	0.993	1.010
**pSHP2B**	1.078	0.970	1.198
**pCD19B**	0.970	0.913	1.030
**pJnkB**	1.013	0.996	1.029
**pPLCg2B**	0.968	0.926	1.012
**pErkB**	0.995	0.983	1.007

Association of Predicted Probabilities and Observed Responses			
**Percent Concordant**	83.9	**Somers' D**	0.678
**Percent Discordant**	16.1	**Gamma**	0.678
**Percent Tied**	0.0	**Tau-a**	0.348
**Pairs**	180	**c**	0.839

MFI baseline

The FREQ Procedure

Frequency<br>Percent<br>Row Pct<br>Col Pct

Table 4 of RA by PRAb			
**RA(RA)**	**PRAb**		
	**n**	**y**	**Total**
**n**	37.04	7.41	44.44
	83.33	16.67	
	71.43	15.38	
**y**	14.81	40.74	55.56
	26.67	73.33	

	28.57	84.62	
**Total**	51.85	48.15	100.00

Sensitivity = 73.33%

Specificity = 83.33%
